# Multicenter retrospective genomic characterization of carbapenemase-producing *Acinetobacter baumannii* isolates from Jiangxi patients 2021–2022: identification of a novel international clone, IC11

**DOI:** 10.1128/msphere.00276-24

**Published:** 2024-06-04

**Authors:** An Xu, Min Li, Yaping Hang, Lingbing Zeng, Xuefei Zhang, Yiyi Hu, Qinglan Guo, Minggui Wang

**Affiliations:** 1 Institute of Antibiotics, Huashan Hospital, Fudan University, Shanghai, China; 2 Key Laboratory of Clinical Pharmacology of Antibiotics, National Health Commission of People’s Republic of China, Shanghai, China; 3 Department of Neurology, The Second Affiliated Hospital of Nanchang University, Nanchang, Jiangxi, China; 4 Department of Clinical Laboratory, The Second Affiliated Hospital of Nanchang University, Nanchang, Jiangxi, China; 5 Department of Clinical Laboratory, The First Affiliated Hospital of Nanchang University, Nanchang, Jiangxi, China; University of Wisconsin-Madison, Madison, Wisconsin, USA

**Keywords:** Carbapenem-resistant *Acinetobacter baumannii*, IC2, IC11, *bla*_NDM-1_, *bla*_OXA-23_

## Abstract

This study aimed to characterize carbapenem-resistant *Acinetobacter baumannii* (CRAB) isolates from Jiangxi patients using whole-genome sequencing (WGS). We subjected 100 clinical CRAB strains isolated from the three local largest teaching hospitals to WGS and antimicrobial susceptibility testing. Molecular epidemiology was investigated using multilocus sequence typing, core genome multilocus typing, core genome single-nucleotide polymorphism phylogeny, and pulsed-field gel electrophoresis. The most prevalent acquired carbapenemase was *bla*_OXA-23_, predominant in all isolates (100%). Isolates belonging to the dominating international clone IC2 accounted for 92% of all isolates. International IC11 (ST164^Pas^/ST1418^Ox^) clone was found in an additional 8% (eight isolates), with seven isolates (87.5%) carrying an acquired additional *bla*_NDM-1_ carbapenemase. The *oxa23*-associated Tn*2009*, either alone or in a tandem repeat structure containing four copies of *bla*_OXA-23_, was discovered in 62% (57 isolates) of IC2. The *oxa23*-associated Tn*2006* was identified in 38% (35 isolates) of IC2 and all IC11 isolates. A putative conjugative RP-T1 (formerly RepAci6) plasmid with *bla*_OXA-23_ in Tn*2006* within AbaR4, designated pSRM1.1, was found in IC2 *A. baumannii* strain SRM1. The *bla*_NDM-1_ gene found in seven IC11 isolates was located on a novel Tn*6924*-like transposon, a first-time report in IC11. These findings underscore the significant importance of real-time surveillance to prevent the further spread of CRAB.

**IMPORTANCE:**

Carbapenem-resistant *Acinetobacter baumannii* (CRAB) is notorious for causing difficult-to-treat infections. To elucidate the molecular and clinical epidemiology of CRAB in Jiangxi, clinical CRAB isolates were collected and underwent whole-genome sequencing and antibiotic susceptibility phenotyping. Key findings included the predominance of OXA-23-producing IC2 *A. baumannii*, marked by the emergence of OXA-23 and NDM-1-producing IC11 strains.

## INTRODUCTION

Carbapenem-resistant *Acinetobacter baumannii* (CRAB) has become a significant threat to global public health. High mortality rates are associated with CRAB infections (1, 2). These infections often occur in immunocompromised and elderly patients in hospital intensive care units (ICUs), and there is a severe lack of effective treatment options for CRAB infections (3).

Carbapenem resistance in *A. baumannii* is largely encoded by acquired OXA-type carbapenemases class D β-lactamases [oxacillinases (OXAs)], especially *bla*_OXA-23-like_. Other acquired OXAs such as *bla*_OXA-24-like_, *bla*_OXA-40-like_, *bla*_OXA-58-like_, *bla*_OXA-143-like_, and *bla*_OXA-235-like_ have been detected. Some variants of chromosomally intrinsic OXA-51-like carbapenemase confer carbapenem resistance when overexpressed by the insertion of an upstream IS*Aba1*. Class B metallo-beta-lactamases [imipenemase (IMP), Verona integron-encoded metallo-beta-lactamases (VIM), Seoul imipenemase (SIM), and New Delhi metallo-beta-lactamases (NDM)] are less frequently reported from CRAB isolates, and only rarely class A carbapenemases [*Klebsiella pneumoniae* carbapenemases (KPC) and Guiana extended-spectrum beta-lactamase (GES)] are identified (4).

Multilocus sequence typing (MLST) schemes are still widely used to investigate the molecular epidemiology and outbreaks of *A. baumannii* (5, 6). The clonal complexes of clustered MLST sequence types (STs) are now corresponding to the international clones (ICs) (7). The assignment to ICs has become an established criterion for classifying *A. baumannii* isolates in an epidemiological context. To date, IC1–11 *A. baumannii* isolates have been described, the most widespread of which is IC2 commonly harboring the acquired carbapenemase OXA-23 (8).

Since the advent of relatively cheap whole-genome sequencing (WGS), this technique has become the ultimate approach to studying the dissemination of many bacterial pathogens. WGS has proven to be extremely useful, offering a higher resolution than the two MLST schemes in the case of *A. baumannii*, where the very dynamic nature of its genome renders MLST typing faulty (7, 9). Although the use of single-nucleotide polymorphism (SNP) typing gives the highest resolution, core genome multilocus typing (cgMLST) provides a more stable nomenclature and results that are easier to share with others, especially considering the very closely related isolates from the same place or strains at very short periods of time (10).

WGS has been used to study the spread of clones of *A. baumannii* at a national level and even the continental level. Over the last years, in some countries, such as the USA, Brazil, Lebanon, Malaysia, United Kingdom, Vietnam, and some parts of China, WGS has been used to analyze clone diversity within and between hospital settings (3, 7, 11–13). However, there is a significant lack of epidemiological and genomic data about different lineages causing CRAB infections in a single hospital setting in Jiangxi province mainly due to limited research infrastructure and resources.

The goal of our study was to elucidate the contemporary population structure of clinical CRAB isolated from patients at the three largest tertiary teaching hospitals in Jiangxi province and the mechanisms of carbapenem resistance. WGS was applied to extract ST^OX^, ST^Pas^, cgMLST, acquired carbapenemase genes and their mobile genetic elements, and capsule locus K as well as outer core locus (OCL) from CRAB isolates (2021–2022), along with epidemiological data and antimicrobial susceptibility testing.

## RESULTS

### Clinical characteristics and antimicrobial susceptibility of clinical CRAB isolates

**Fig 1:**
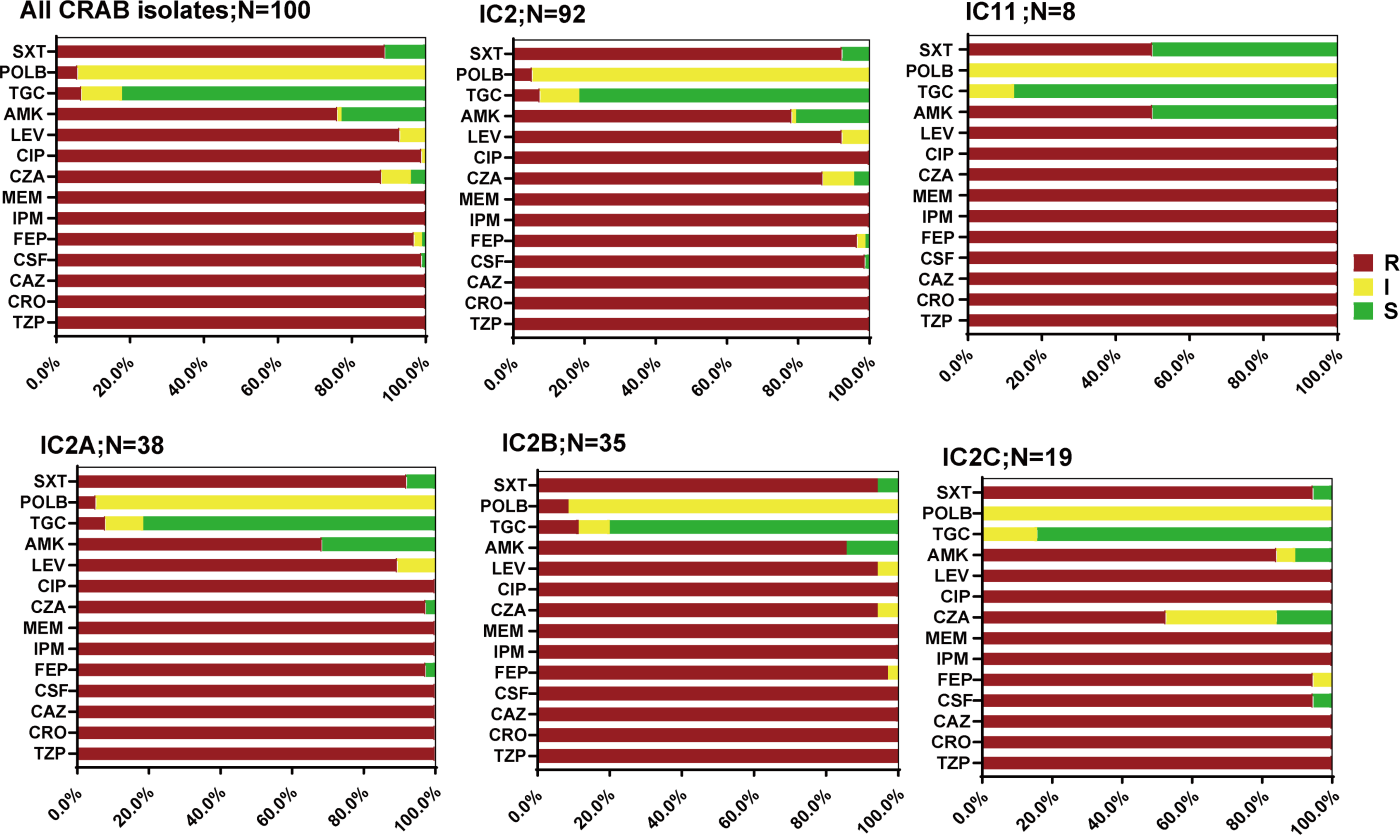
Antimicrobial susceptibility profiles of CRAB isolates. Antimicrobial susceptibilities of 100 tested isolates were determined with broth microdilution. MEM, meropenem; CRO, ceftriaxone; CAZ, ceftazidime; FEP, cefepime; CSF, cefoperazone–sulbactam; TZP, piperacillin–tazobactam; CZA, ceftazidime–avibactam; ATM, aztreonam; CIP, ciprofloxacin; LEV, levofloxacin; AMK, amikacin; TGC, tigecycline; POLB, polymyxin B; SXT, trimethoprim–sulfamethoxazole. Interpretations of susceptibilities were assigned according to the Clinical and Laboratory Standards Institute (CLSI M100-ED31) guideline. Tigecycline susceptibility was followed based on FDA Enterobacterales breakpoints. S, susceptible; I, intermediate; R, resistant.

A total of 100 non-duplicate CRAB strains were collected, with one lacking clinical ward information and two missing specimen origin data. Out of 98 isolates with specified sources, 82.5% originated from the respiratory tract, followed by 7.1% from blood (Table 1). Of the 99 isolates with known origins, 60.6% were derived from ICUs (Table S2). Rates of resistance to all the 14 tested antimicrobials were high among the isolates (Fig. 1; Table S3). Among the 100 tested isolates, 79.0% were classified as extensively drug-resistant *Acinetobacter baumannii* (XDRAB), defined as exhibiting non-susceptibility to at least one agent in all but two or fewer antimicrobial categories (14–16). Rates of tigecycline and polymyxin B resistance were relatively low, at 7.0% (7/100) and 5% (5/100), respectively. Furthermore, 11.0% (11/100) of the isolates showed intermediate resistance to tigecycline.

**TABLE 1:** Culture source distribution of CRAB isolates^*a*^

Sublineage	cgMLST	No. (%)									
		Total (*n* = 100)	Respiratory (*n* = 81)	Blood (*n* = 7)	Abdominal (*n* = 2)	Abscess (*n* = 1)	Bile (*n* = 2)	CSF (*n* = 2)	Wound (*n* = 1)	Urine (*n* = 1)	NA (*n* = 3)
IC2A	cgST596	31 (31)	20 (66)	4 (13)	1 (3)	1 (3)	2 (6)	1 (3)			2 (6)
	cgST668	7 (7)	7 (100)								
IC2B	cgST906	35 (35)	30 (85)	2 (6)	1 (3)			1 (3)		1 (3)	
IC2C	cgST923	14 (14)	13 (93)						1 (7)		
	cgST661	2 (2)	2 (100)								
	cgST909	3 (3)	2 (67)	1 (33)							
IC11	cgST1101	8 (8)	7 (88)								1 (12)

^*a*^ NA, unknown origin; CSF, cerebrospinal fluid.

**TABLE 2:** Study site distribution of CRAB isolates

Sublineage	cgMLST	No. (100%)			
		Total (*n* = 100)	YFY (*n* = 29)	EFY (*n* = 34)	SRM (*n* = 37)
IC2A	cgST596	31 (31)	6 (21)	14 (41)	11 (30)
	cgST668	7 (7)	4 (14)	3 (8)	
IC2B	cgST906	35 (35)	11 (38)	6 (18)	18 (49)
IC2C	cgST923	14 (14)	4 (14)	8 (24)	2 (5)
	cgST661	2 (2)			2 (5)
	cgST909	3 (3)	1 (3)	2 (6)	
IC11	cgST1101	8 (8)	3 (10)	1 (3)	4 (11)

### Dominance of IC2 and emergence of IC11 within CRAB population across multiple hospitals

**Fig 2:**
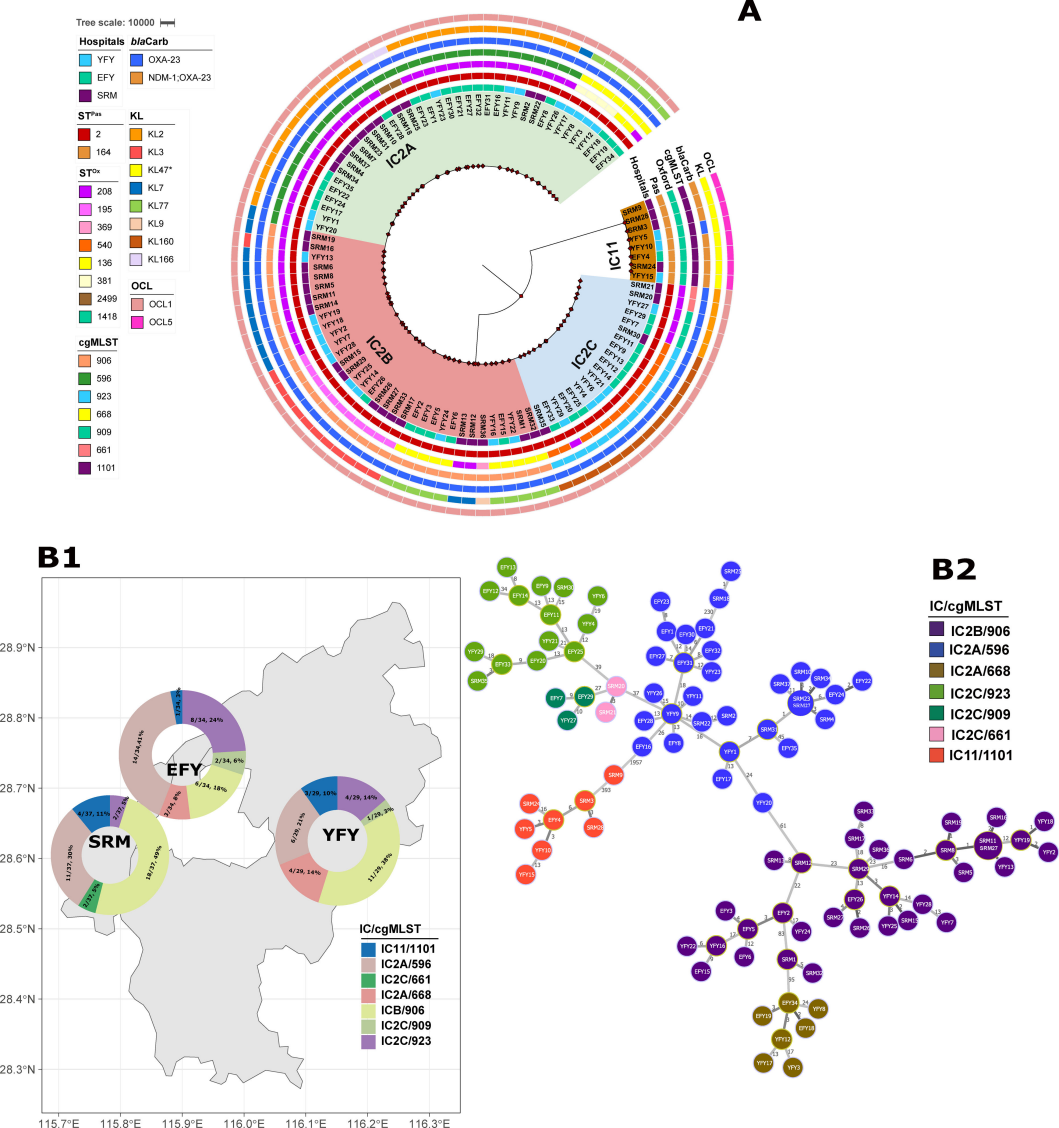
Phylogeny of A. *baumannii* with distribution of isolates and international clones. (**A**) The midpoint rooted phylogeny was constructed from SNPs in the core genomes of all isolates using RAxML. The phylogeny is annotated based on the study site of isolation, IC, Oxford ST, core genome MLST (determined by cgMLSTFinder v1.2), acquired carbapenemase, capsular polysaccharide K locus (KL), and lipooligosaccharide OCL. Branches are shaded by lineages and sublineages described in the text. EFY, the Second Affiliated Hospital of Nanchang University; YFY, the First Affiliated Hospital of Nanchang University; SRM, Jiangxi Provincial People’s Hospital. (**B1**) Geographical distribution of cgMLST of the 100 CRAB isolates. The maps were drawn using the R package mapchina (https://github.com/xmc811/mapchina), and the data source was derived from https://www.openstreetmap.org. (**B2**) Minimum spanning tree constructed on the basis of cgMLST allelic genes of 100 clinical CRAB isolates. Each circle depicts an allelic profile based on sequence analysis of 2,133 cgMLST genes. Colors of the circles denote different cgMLST types. Closely related genotypes (less than 10 alleles difference) are shaded in the same node. The length of the connecting lines represents the number of target genes with different alleles.

According to the recently established international clones (IC1–11), a total of 100 isolates could be assigned to the two ICs, IC2 and IC11. A whole-genome phylogeny based on SNPs in the core genomes of all isolates is shown in Fig. 2A. We defined a SNP cutoff based on the observed SNP distribution to cluster study isolates into clearly separated clonal lineages and sublineages, which were then compared to established ICs. A cutoff of 46,000 SNPs differentiated CRAB lineages belonging to IC2 and IC11. A cutoff of 220 SNPs further defined major sublineages within the ICs (Table S1). Three sublineages within IC2 with various degrees of heterogeneity are showed in Fig. 2A and presented in Table S1. The geographical distribution of CRAB isolates by study hospital, culture source distribution, and ward distribution is illustrated in Fig. 2; Tables 1 and 2; Table S2.

**Fig 3:**
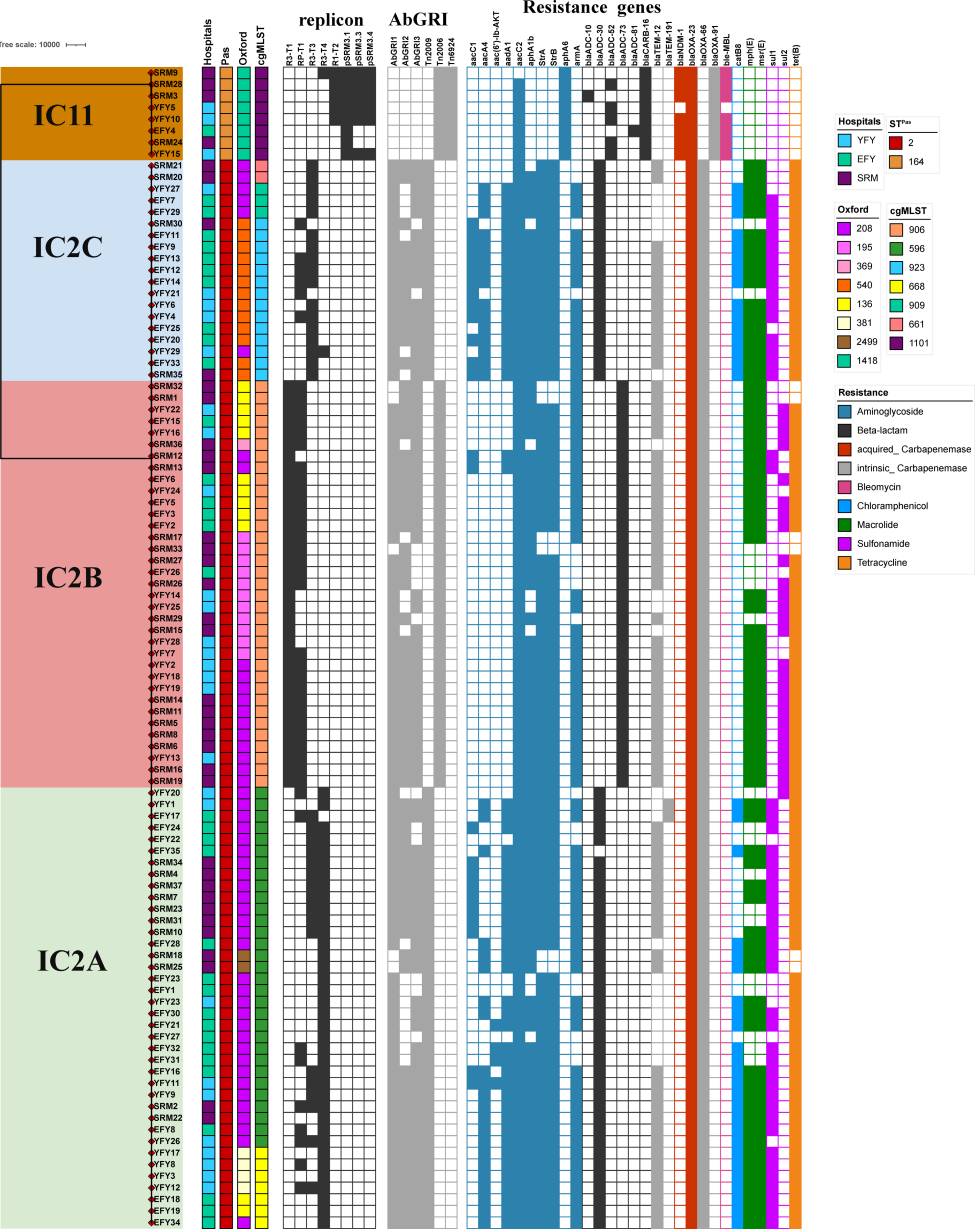
Genetic characteristics of OXA-23-producing IC2 and IC11 clinical CRAB originated from Jiangxi region. The phylogeny is annotated based on Pasteur ST, Oxford ST, cgMLST, and study hospital of isolation. The presence of plasmids and plasmid types is shown in black, and their absence is in white; pSRM1.2 and pYFY24.2 have identical genome sequences, represented by pSRM1.2; pYFY21.1 and pYFY24.1 have nearly identical genome sequences illustrated by pYFY21.1; pYFY3.1 and pSRM25.1 contain identical genome sequences, depicted by pSRM25.1. The presence of genomic resistance islands (RIs) and resistance transposons is shown in gray, and their absence is in white.

IC2 constituted the largest and most widely spread clonal lineage with isolates (92%) distributed over all participating hospitals. Novel IC11 was as widespread over all study sites as IC2, but with a much lower prevalence, accounting for only eight isolates (8%) (Fig. 2; Table 2).

cgMLST sequence types (cgSTs) were determined by CGE cgMLSTFinder based on the pubMLST database with 2,133 alleles. Seven different cgSTs were assigned to the 100 available isolates (Fig. 2B2). IC2 sublineage A (IC2A) comprised two CGE cgSTs, including cgST596 and cgST668 (Tables 1 and 2; Tables S1 and S2). IC2 sublineage B (IC2B) corresponded to cgS906. The majority of IC2 sublineage C (IC2C) isolates belonged to cgST923, in addition to cgST909 and cgST661 (Table 2). IC11 belonged to cgST1101. The cgST distributions of CRAB isolates including study hospital, culture sources, and isolation ward are depicted in Tables 1 and 2; Table S2; Fig. 2B1. cgST596, cgST906, and cgST923 are the three major dominant lineages widely distributed across all three hospitals, but IC11/cgST1101 is widespread with low prevalence (Table 2; Fig. 2B1). The body site distribution of cgSTs was similar to one another; most cgSTs are characterized by the predominance in the respiratory tract, with the exception of cgST596 and cgST906 distributed in a variety of isolation sources (Table 1). All cgSTs were primarily found in hospital ICUs, but cgST596, cgST906, and cgST923 were also present in different clinical wards (Table S2).

We searched the OCL and capsular polysaccharide KL types of virulence contributors (Fig. 2A). The OCL1 capsule was predominant in IC2 (100% of isolates), but seven different KL variants were present, with KL2, KL77, KL7, KL160, and KL3 representing more than 80% of the types (34.8%, 18.5%, 15.2%, 15.2%, and 13.0%, respectively). For each IC2 sublineage, in IC2A, KL2 predominated 71%, and the KL variants for IC2B were KL3, KL7, and KL77 (34.3%, 34.3%, and 28.6%, respectively); in IC2C, KL160 was predominant (73.7%). In IC11, OCL5 was predominant (100%) and KL47* variant was present (100%). Overall, we found eight KL types, with KL2, KL77, KL160, KL7, and KL3 representing about 88% of the types (31%, 17%, 14%, 14%, and 12%, respectively). In contrast, there were only two different O capsule types; however, these were dominated by OCL1 due to its presence in the predominant international clone IC2.

### Characterization of the resistance transposons, genomic resistance islands, and plasmids among clinical CRAB population

An acquired carbapenemase gene was identified in all tested isolates across all study sites (Fig. 2A and 3). The most frequently encountered acquired carbapenemase was *bla*_OXA-23_, present in all tested isolates. Seven isolates (7%) harbored an additional *bla*_NDM-1_; interestingly, *bla*_NDM-1_ was restricted to IC11 isolates (87.5%). Two *oxa23*-associated transposons, Tn*2006* and Tn*2009*, were identified (Fig. 3). *oxa23*-containing Tn*2006* was confined to IC2B and IC11, and *oxa23*-containing Tn*2009* was present only in IC2A and IC2C isolates. A novel transposon associated with *bla*_NDM-1_, designated Tn*6924-*like, was found in IC11 isolates (100%).

**Fig 4:**
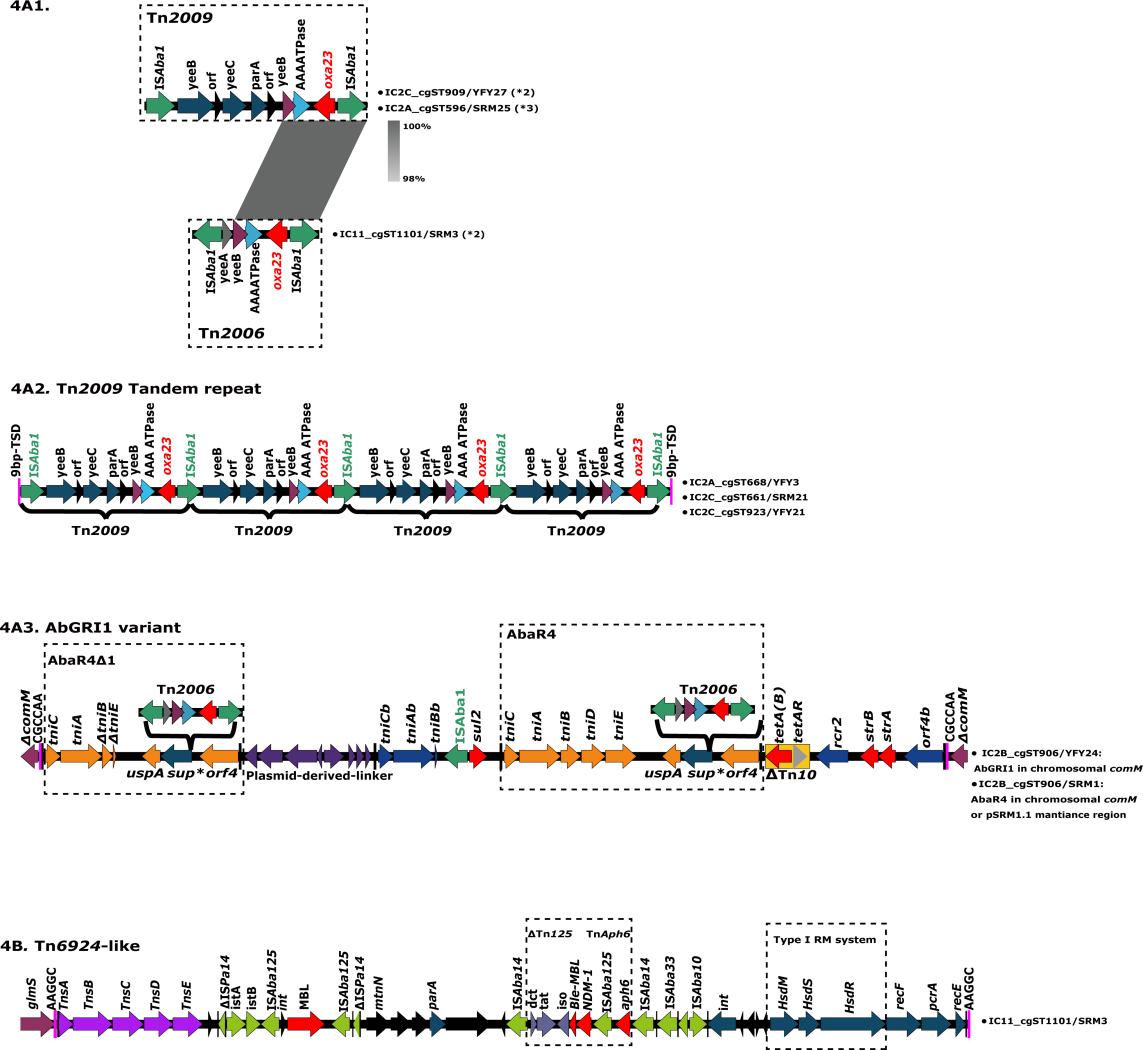
Genetic context of acquired *bla*_OXA-23_ and *bla*_NDM-1_. The orientation and extent of genes are indicated by horizontal arrows. Inverted repeats and TSDs are shown as black and violaceous vertical bars, respectively. (A1) Structure comparison of Tn*2009* and Tn*2006* carrying the *bla*_OXA-23_ gene. *2 or *3 indicates that Tn*2009* or Tn*2006* move independently into two or three chromosomal locations. (A2) Genetic structure of a Tn*2009* tandem repeat structure with four copies of *bla*_OXA-23_ (located in the chromosomes of isolates IC2A_cgST668/YFY3, IC2C_cgST661/SRM21, and IC2C_cgST923/YFY21). (A3) Tn*2006* within AbaR4 and AbaR4 derivative as part of AbGRI1 variant in the chromosomal *comM* of strain IC2B_cgST906/YFY24. Orange, dark blue, and purple denote Tn*6022*, Tn*6172,* and the linker elements, respectively. (B) *bla*_NDM-1_-containing Tn*6924*-like transposon structure identified in strain IC11_cgST1101/SRM3 in this study. Comparison of Tn*6924* found in Cl300 (GenBank accession no. CP082952) and 11W359501 (GenBank accession no. CP041035). Tn*7* transposition genes are colored purple, antibiotic resistance genes red, and insertion sequence green.

To characterize the genetic context of the acquired *bla*_OXA-23_ and *bla*_NDM-1_ genes in each sublineage, high-quality complete genomes of eight representatives with distinct cgSTs from each sublineage were analyzed (Table S4; Fig. 4). The genetic environment of *bla*_OXA-23_ is illustrated in (Fig. 4A1 through A3). In IC2A and IC2C, *bla*_OXA-23_-containing Tn*2009* was either found alone or in a tandem repeat structure with four copies of *bla*_OXA-23_, which upon integration into the chromosome generated diverse 9 bp target site duplications (TSDs). In IC2B, *bla*_OXA-23_-containing Tn*2006* was commonly present within AbaR4 or its derivatives, targeting the chromosomal *comM* gene or disrupting a repAci6 plasmid backbone. The AbGRI1 variant harboring AbaR4 also targeted the chromosomal *comM* gene. In the IC11 strain (SRM3), two copies of *bla*_OXA-23_-containing Tn*2006* transposed into two distinct chromosomal locations, producing different 9 bp TSDs. One Tn*2006* was located near an intact prophage region, generating a TSD of AAAATTGAG, while the other Tn*2006* integrated into a gene encoding an efflux RND transporter permease, with a TSD of AAAATATCG. The genetic structure of the NDM-1-associated Tn*6924-*like transposon is depicted in Fig. 4B. This transposon, a 31.5 kb member of the Tn*7* superfamily, confers resistance to carbapenem and amikacin. It comprises multiple insertion sequences, a truncated Tn*125* transposon carrying a copy of the *bla*_NDM-1_ gene, an *aphA6* Tn*aphA6*-like transposon, and a type I restriction methylation (R1M1S1) system. Additionally, it generates a 5 bp TSD upon insertion into the 3′ end of chromosomal *glmS* (17–19).

The variants of AbGRI1, AbGRI2, and AbGRI3 RIs are present in most IC2 isolates, while no known RIs were identified in IC11 isolates (Fig. 3). Both AbGRI2 and AbGRI3 are IS*26*-mediated RIs. The AbGRI1-like RI typically confers resistance to sulfamethoxazole (*sul2*), streptomycin (*strA-strB*), tetracycline [*tetA*(*B*)], and carbapenem (*bla*_OXA-23_), targeting the chromosomal *comM* locus. The AbGRI3-like RI commonly inserts within the *atr* locus, encoding a putative GNAT family N-acetyltransferase, and contains resistance genes for aminoglycoside (*armA*), macrolide (*msrE-mphE*), gentamicin/tobramycin (*aacA4*), chloramphenicol (*catB8*), streptomycin (*aadA1*), sulfonamide (*sul1*), and kanamycin (*aphA1b*). The AbGRI2-like RI often carries genes for resistance to cephalosporin (*bla*_TEM_), gentamicin (*aacC1*), streptomycin (*aadA1*), sulfonamide (*sul1*), and kanamycin (*aphA1b*).

We detected five known plasmid replicon types (20–22) and three novel types in our CRAB collection (Table S4; Fig. 3). The distributions of plasmid replicons within each IC2 sublineage showed that R3-T4 (97.3%), R3-T3 (44.7%), and RP-T1 (23.7%) replicons were found in a total of 38 IC2A isolates, especially, in IC2B (35 isolates), most co-harbored RP-T1 (100%) and R3-T1 (82.9%) plasmids (Fig. 3). In IC2C of 19 isolates, R3-T3 (78.9%), RP-T1 (31.6%), and R3-T4 (5.3%) were detected. The replicons in IC11 were R3-T4 (one isolate), R1-T2 (five isolates), pSRM3.1 (eight isolates), pSRM3.3 (six isolates), and pSRM3.4 (six isolates) (Fig. 3). R3-T1, R3-T4, R1-T2, pSRM3.1, pSRM3.3, and pSRM3.4 replicons were associated with small plasmids (Table S4). The RP-T1 plasmids (72–86 kb) could be further differentiated into two plasmid backbones, represented by pYFY21.1 (nearly identical in sequence to pYFY24.1) and pSRM1.1 (characterized by the insertion of a *bla*_OXA-23_-containing Tn*2006* within AbaR4 upstream of the maintenance region). The R3-T3 plasmids (~112 kb) are represented by pYFY27.1 (showing approximately 101,306 bp alignment with pSRM21.1 and having 85.54% nucleotide identity). The R3-T1 plasmids (8.73 kb) are represented by pSRM1.2 and pYFY24.2 with identical sequences. The R3-T4 plasmids (11.19 kb) are represented by identical pYFY3.1 and pSRM25.1 (76.9% nucleotide sequence identity with pYFY27.2). The R1-T2 plasmid (2.3 kb) is represented by pSRM3.2. pSRM3.1, pSRM3.3, and pSRM3.4 do not contain a recognizable *rep* gene and, therefore, cannot be assigned to a Rep group according to established typing protocols for *Acinetobacter* plasmids (20–22).

### Emergence of the international IC11 clone co-harboring chromosomal OXA-23 and NDM-1 in China

The novel international IC11 (ST164^Pas^/ ST1418^Ox^) clone was also sporadically recognized in this study, accounting for 8% of all isolates, seven (87.5%) of which contained both *bla*_NDM-1_ and *bla*_OXA-23_-acquired carbapenemase genes. IC11 isolates were more likely to have meropenem minimum inhibitory concentrations (MICs) ≥ 64 mg/L and imipenem MICs ≥ 128 mg/L than IC2 isolates (Table S3). IC11 isolates (100%) had meropenem MICs of ≥64 mg/L and imipenem MICs of ≥128 mg/L, compared to 61 (66.3%) and 11 (11.9%) out of 92 IC2 isolates, respectively.

**Fig 5:**
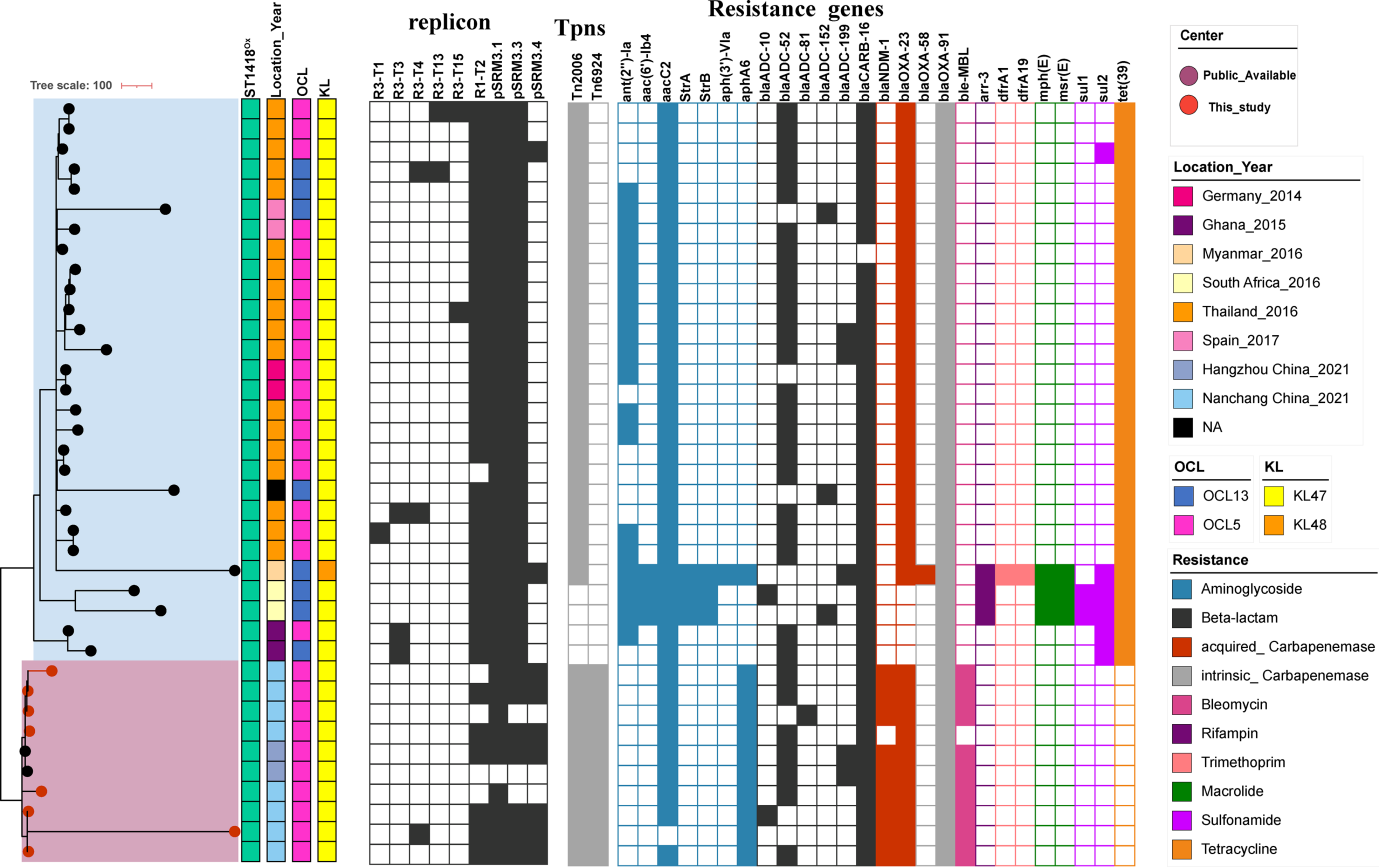
A global view of IC11 (ST164^Pas^/ST1418^Ox^) *Acinetobacter baumannii* based on WGS. Core genome phylogeny is linked with country, collection date, study center, capsular polysaccharide KL, lipooligosaccharide OCL, and antimicrobial resistance genes. The highlighted boxes denote the substructure within ST1418^Ox^ (Thailand subclade and China subclade). The presence of plasmid types is shown in black, and absence is in white; the presence of resistance transposons is shown in gray, and absence is in white. Abbreviations: NA, not applicable; Tpns, transposon.

Since IC11 has been rarely characterized and often found sporadically worldwide (8, 23). We, therefore, reviewed our findings in the context of IC11 (ST64^Pas^/ST1418^Ox^) by incorporating sequences from Pathogenwatch and NCBI into our own assemblies (Fig. 5; Table S5). These isolates were collected between 2014 and 2021 from Germany (2014), Ghana in West Africa (2015), Myanmar (2016), South Africa (2016), Thailand (2016), Spain (2017), and Hangzhou and Nanchang, China (2021). The global phylogenetic analysis showed that IC11 appears to have originated in Thailand, subsequently evolving into two clades. Most of the isolates from Thailand were grouped in one clade, while those from China were clustered into another. A total of 34 IC11 isolates (89.5%) carried an acquired carbapenemase gene. As IC2, the majority of IC11 isolates (70.6%) carried *bla*_OXA-23_. Nine isolates (26.5%) from China carried *bla*_OXA-23_ in addition to the *bla*_NDM-1_. One isolate co-harbored *bla*_OXA-23_ and *bla*_OXA-58_. Notably, the *bla*_OXA-23_ gene was only located on Tn*2006,* and the *bla*_NDM-1_ gene was within Tn*6294-*like transposon. R1-T2, pSRM3.1, and pSRM3.3 were the three most frequent replicons in IC11. No resistance genes were identified on these plasmids. No known RIs were found in IC11 isolates. KL47 was predominant in IC11 (97.4% of 38 isolates). The OCL variants for IC11 were OCL5 and OCL13 (78.9% and 21.1%, respectively).

## DISCUSSION

Due to the limited availability of detailed data on clinical CRAB populations in Jiangxi province, we conducted a genome-based survey on CRAB strains obtained from the three largest tertiary teaching hospitals in this region. As expected, the majority of strains analyzed (60.6%) were collected from hospital ICUs, and they commonly (82.5%) occurred in the patients’ respiratory tracts (1, 3, 11). Consistent with previous reports, most strains (80%) were phenotypically XDRAB (3, 15). However, of great concern, the CRAB strains analyzed in this study exhibited higher resistance rates to polymyxin B (5%) and tigecycline (7%) compared to the results reported in China’s ICUs in 2020 (3). Investigating the underlying resistance mechanisms for polymyxin B and tigecycline is beyond the scope of this work; further research on these aspects will be conducted in the future.

IC2 was by far the most frequent clonal lineage and was spread worldwide, although new lineages have become common in some parts of the world and are often associated with the dissemination of *bla*_OXA-23_ gene among CRAB (9). In the current study, IC2 predominated in our CRAB population, accounting for 92%, consistent with previous findings, and was followed by IC11 (8%).

ST164^Pas^ *A. baumannii* has spread globally over the past decade, though with low prevalence. It has, thus, been recognized as an international clone, recently proposed to be named IC11 (8, 23–27). However, to date, we still lack sufficient IC11 data in China. In this study, the sporadic occurrence of international clone IC11 provides an update on the molecular epidemiology of CRAB. More importantly, the emergence of NDM-1 and OXA-23-producing IC11 isolates is of great concern for healthcare settings, requiring real-time and effective surveillance to prevent further dissemination. NDM-1-producing *A. baumannii* isolates are uncommon. Many studies found that NDM-1-positive CRAB are IC9 (ST85^Pas^) from the Middle East and sporadically in Germany and Denmark (8, 27). A recent study by Zafer et al. described an NDM-1-producing IC11 (ST164^Pas^/ST1418^Ox^) *A. baumannii* from Cairo, Egypt (27). Although OXA-23-/NDM-1-producing *A. baumannii* isolates are relatively rare, Miltgen et al. (28) reported an outbreak of such IC1 *A. baumannii* without special phenotype in the Southwest Indian Ocean Area. Similarly, Hansen et al. (8) identified six similar isolates in Denmark, comprising five IC2 and one IC1. Notably, Zhao et al. (23) first reported an IC11 (ST164^Pas^/ST1418^Ox^) strain collected from an ICU patient in Hangzhou, marked by three copies of OXA-23 and one copy of NDM-1 inserted into the chromosome via different composite transposons. In accordance with previous studies, our comprehensive genomic analysis suggests that the IC11 (ST164^Pas^/ST1418^Ox^) *A. baumannii* strain has spread worldwide over the last decade (8, 24, 27), but it remains at low prevalence and requires further surveillance due to the limited number of available genomes. Also, in the present analysis, we identified two distinct IC11 clades, one cluster characterized by OXA-23-producing strains primarily from Thailand and another comprising OXA-23-/NDM-1-producing strains originating from China. This provides important evidence for empirical clinical treatment of CRAB infections in healthcare settings in China.

Recent studies have revealed that traditional seven-loci schemes (Oxford and Pasteur) and pulsed-field gel electrophoresis typing (our data also showed in Fig. S1) fail to accurately depict the relationships among *A. baumannii*, primarily due to the high levels of recombination events (29–31). In our study, we achieved finer resolution in CRAB population analysis using core genome single-nucleotide polymorphisms and cgMLST typing, thereby uncovering detailed genetic patterns. Variations in geographic distribution, antimicrobial susceptibility, and mobile genetic elements (RIs, plasmids, and transposons) were observed between IC2 (with its distinct sublineages) and IC11. Previous findings indicated that cgSTs displayed a close association with transposon types among IC2 isolates (3, 11). In this study, Tn*2006* was present in IC2B, corresponding to cgST906, and in IC11, classified under cgST1101. The RP-T1 (formerly RepAci6) pSRM1.1 found in IC2B isolates was indicative of playing a great role in the dissemination of *bla*_OXA-23_ between species, given that this plasmid contained a copy of *bla*_OXA-23_ in Tn*2006* within AbaR4 inserted into the plasmid maintenance region, but with a complete conjugative region. RepAci6 plasmids were known to facilitate the spread of *oxa23* gene in IC1 and IC2 (4). Some RepAci6 plasmids have been reported to be conjugative and include a complete set of genes for conjugation and could be a helper plasmid to facilitate a *bla*_OXA-23_-carrying RepAci1 plasmid mobilization (4, 32). Additionally, Tn*2009*, frequently found in IC2A and IC2C strains, could be represented in a tandem repeat structure with four copies of *bla*_OXA-23_, which has been poorly reported (4, 9, 33, 34). Few studies demonstrated that the formation of a Tn*2009* tandem repeat circular intermediate, assisted by the IS*Aba1* element, suggests a novel mechanism for *bla*_OXA-23_ translocation, warranting further investigation (9). The acquisition of *bla*_NDM-1_ by a novel Tn*6924*-like was firstly reported in IC11 (ST164^Pas^/ST1418^Ox^) *A. baumannii,* and its contribution to the spread of *bla*_NDM1_ shall be more concerning (17, 19, 23).

Our study has several limitations. This analysis did not encompass all hospitals in this region, suggesting that the clinical and genome epidemiology of CRAB may differ. Another limitation of this study was that we did not determine the impact of *bla*_OXA-23_ copy number on phenotypic susceptibility.

In summary, IC2 has predominated the OXA-23-producing CRAB population in this region, marked by the emergence of the rare IC11 (ST164^Pas^/ST1418^OX^) lineage co-harboring *bla*_OXA-23_ and *bla*_NDM-1_. This discovery highlights the critical importance of ongoing surveillance to prevent further dissemination.

## MATERIALS AND METHODS

### Strain collection

A total of 100 non-duplicate CRAB strains were collected by three clinical laboratories at the three local distinct largest tertiary teaching hospitals in Jiangxi province. All strains were recovered from patients admitted to the hospitals between February 2021 and February 2022. The study was approved by the Ethics Committee of the Second Affiliated Hospital of Nanchang University. As we conducted a retrospective study, it was exempt from the requirement for informed consent.

### Species identification and antimicrobial susceptibility testing

*Acinetobacter* strains were identified by matrix-assisted laser desorption/ionization time of flight mass spectrometry (Bruker, Bremen, and Germany). MICs of different antimicrobial agents were determined by the broth microdilution method according to the Clinical and Laboratory Standards Institute (CLSI 2021) guidelines with the reference strain *Escherichia coli* ATCC 25922 being used as the quality control (35). The MIC interpretive breakpoints of tigecycline for *A. baumannii* were determined based on the FDA recommendations for Enterobacteriaceae: susceptible (≤2 µg/mL), intermediate (4 µg/mL), and resistant (≥8 µg/mL) (3, 16, 36).

### Whole-genome sequencing and analysis

Genomic DNA of all isolates was extracted using the TIANamp Bacteria DNA kit (Tiangen Biochemical Technology, Beijing) following the manufacturer’s instructions and was subjected to WGS described below. A total of 100 CRAB isolates were sequenced using a HiSeq X Ten Sequencer (Illumina, San Diego, CA, USA), with 150  bp paired-end short reads and 200× coverage (Table S6). Of the 100 isolates, eight isolates were also subjected to long-read sequencing using a MinION Sequencer (Nanopore; Oxford, United Kingdom). The obtained paired-end Illumina reads were assembled *de novo* with the use of the SPAdes v3.13 (37). Both short and long reads of the additional eight isolates were utilized to generate a *de novo* hybrid assembly using Canu v1.8 and Pilon v1.24 (38). Genomes were annotated using Prokka v.12-beta and NCBI Prokaryotic Genome Annotation Pipeline (39, 40). Short reads were mapped to reference genomes of respective international clones using Snippy v.4.6.0 (IC2, A9844, CP102580.1; IC11, XH1935, CP088894.1). Recombination was identified and removed by Gubbins v.3.3.0 (41), and the resulting alignment was used for tree estimation using RAxML v8.2.12 with GTRGAMMA model and 100 rapid bootstrap replicates (42). In order to understand the epidemiology of IC11 (ST164^Pas^/ST1418^OX^) clone from a global perspective, we analyzed the IC11 (ST164^Pas^/ST1418^OX^) sequences by investigating 28 assemblies available through Pathogenwatch (https://pathogen.watch/) and only two genomes deposited in the NCBI database, from a variety of countries (Table S5). Assemblies and genomes were downloaded in December 2023 through their websites and included with our own assemblies. SNP distances were calculated from the Gubbins-filtered polymorphic sites file using SNP-dists v0.6.3. Visualization of trees was carried out using Figtree v1.4.4 and iTOL v6 (11).

### Molecular epidemiology

Seven-loci MLST (Pasteur and Oxford) schemes and cgSTs were derived from genome assemblies of all isolates. MLSTs were performed *in silico* with the MLST v2.23.0 program (http://github.com/tseemann/mlst). cgSTs were determined using cgMLSTFinder v1.2 (http://www.genomicepidemiology.org/) and were further clustered using PHYLOViZ v.2.0.

### Identification of resistance genes, plasmid replicons, and resistance islands

Resistance genes were identified from genome assemblies of all isolates using ABRicate v1.0.1 with the ResFinder database (https://github.com/tseemann/abricate). Replicons were identified based on the latest replication initiation (Rep) protein gene typing scheme developed for *A. baumannii* plasmids (12, 20–22). The presence of genomic resistance islands and *oxa23*-associated transposons in *A. baumannii* were investigated using BLASTn, as described previously (4, 43, 44). The identification of Tn*6294* transposon in IC11 isolates was conducted using BLASTn and ISFinder (18, 19, 23).

## Data Availability

Illumina raw sequence reads and eight complete genomes have been deposited in the NCBI database under BioProject number PRJNA992789, accession numbers SAMN36374389 to SAMN36374928.

## SUPPLEMENTAL MATERIAL

Supplemental material msphere.00276-24-s0001.docx Supplemental tables and figure.

ASM does not own the copyrights to Supplemental Material that may be linked to, or accessed through, an article. The authors have granted ASM a non-exclusive, world-wide license to publish the Supplemental Material files. Please contact the corresponding author directly for reuse.
