# Supplementary material for: Multicenter retrospective genomic characterization of carbapenemase-producing Acinetobacter baumannii isolates from Jiangxi patients 2021–2022: identification of a novel international clone, IC11
Source: mSphere. 2024 Jun 4;9(6):e00276-24. doi: 10.1128/msphere.00276-24 (PMC11332331; doi:10.1128/msphere.00276-24)
Supplement: Supplemental material — Supplemental tables and figure. [file msphere.00276-24-s0001.docx]

**SUPPLEMENTAL MATERIAL**

Table S1. Pairwise core genome SNP comparisons calculated from total core genome of 100 isolates

Table S2. Ward origin distribution of CRAB isolates

Table S3. Antimicrobial susceptibility profiles of 100 non-duplicate clinical CRAB isolates

Table S4. Plasmids within each CRAB sublineage

Table S5. Characteristics of IC11 (ST164^Pas^/ST1418^Ox^) *A. baumannii*

Table S6. Characteristics of 92 OXA-23-producing IC2 *A. baumannii* in this study

Figure S1. Pulsed-field gel electrophoresis (PFGE) characteristics of 100 clinical CRAB isolates

**Table S1. Pairwise core genome SNP comparisons calculated from total core genome of 100 isolates.** NA, not applicable, too few isolates for calculation

| **IC <46,000 SNPs** | **Sublineage**  **<220 SNPs** | **cgMLST** | **ST^Pas^** | **ST^OX^** | **n** | **Median SNPs**  **(range)** |
| --- | --- | --- | --- | --- | --- | --- |
| IC2 | IC2A | 596 | 2 | 208 | 29 | 1.6 (1-61) |
|  |  |  | 2 | 2499 | 2 |  |
|  |  | 668 | 2 | 136 | 1 |  |
|  |  |  | 2 | 208 | 2 |  |
|  |  |  | 2 | 381 | 4 |  |
|  | IC2B | 906 | 2 | 208 | 13 | 1.6 (1-55) |
|  |  |  | 2 | 195 | 11 |  |
|  |  |  | 2 | 136 | 10 |  |
|  |  |  | 2 | 369 | 1 |  |
|  | IC2C | 661 | 2 | 208 | 2 | 6.9 (2-131) |
|  |  | 909 | 2 | 208 | 3 |  |
|  |  | 923 | 2 | 208 | 1 |  |
|  |  |  | 2 | 540 | 13 |  |
| IC11 | D | 1101 | 164 | 1418 | 7 | 26 (1-31) |
|  | E | 1101 | 164 | 1418 | 1 | NA |

**Table S2. Ward origin distribution of CRAB isolates**

ICU, Intensive Care Unit; CM, Cardiovascular Medicine; CMS, Cardiac and macrovascular surgery; GIS, Gastrointestinal Surgery; CTS, Department of Cardiothoracic Surgery; HS, Hepatobiliary Surgery; Nep, Nephrology; NL, Neurology; NS, Neurosurgery; PCCM, Pulmonary and Critical Care Medicine; EM, Emergency Medicine; RH, Rehabilitation; ULS, Urology; OD, Oncology; ORTH, Orthopedic; GS, General Surgery. NA, unknown. The total 100CRAB isolates, 60 strains collected from ICU, 20 strains from surgery wards and 20 strains from internal Medicine

| **Sublineage** | **cgMLST** | **No. (%)** | | | | | | | | | | | | | | | | | |
| --- | --- | --- | --- | --- | --- | --- | --- | --- | --- | --- | --- | --- | --- | --- | --- | --- | --- | --- | --- |
|  |  | **Total**  **(n= 100)** | **ICU**  **(n= 60)** | **CM**  **(n= 2)** | **CMS**  **(n= 3)** | **GIS**  **(n= 2)** | **CTS**  **(n= 1)** | **HS**  **(n= 4)** | **Nep**  **(n=2)** | **NL**  **(n= 2)** | **NS**  **(n= 7)** | **PCCM**  **(n= 8)** | **EM**  **(n= 1)** | **RH**  **(n= 2)** | **ULS**  **(n= 1)** | **OD**  **(n= 1)** | **ORTH**  **(n= 1)** | **GS**  **(n= 1)** | **NA**  **(n= 2)** |
| **IC2A** | **cgST596** | 31 (31) | 13 (42) | 1 (3) | 2 (7) | 1 (3) | 1 (3) | 4 (13) | 1 (3) | 1 (3) | 3 (10) | 1 (3) |  |  |  |  |  | 1 (3) | 2 (7) |
|  | **cgST668** | 7 (7) | 5 (72) |  |  |  |  |  |  |  | 1 (14) |  | 1 (14) |  |  |  |  |  |  |
| **IC2B** | **cgST906** | 35 (35) | 24 (69) |  | 1(3) | 1(3) |  |  | 1(3) | 1(3) |  | 5 (14) |  | 1(3) | 1(3) |  |  |  |  |
| **IC2C** | **cgST923** | 14 (14) | 7 (50) |  |  |  |  |  |  |  | 2 (14) | 2 (14) |  | 1 (7) |  | 1 (7) | 1 (7) |  |  |
|  | **cgST661** | 2 (2) | 1 (50) | 1 (50) |  |  |  |  |  |  |  |  |  |  |  |  |  |  |  |
|  | **cgST909** | 3 (3) | 2 (67) |  |  |  |  |  |  |  | 1 (33) |  |  |  |  |  |  |  |  |
| **IC11** | **cgST1101** | 8 (8) | 8 (100) |  |  |  |  |  |  |  |  |  |  |  |  |  |  |  |  |

**Table S3. Antimicrobial susceptibility profiles of 100 non-duplicate clinical CRAB isolates**

The antimicrobial susceptibilities were determined with broth microdilution. IMP, imipenem; MEM, meropenem; CRO ceftriaxone; CAZ, ceftazidime; FEP, Cefepime; CSF, cefoperazone-sulbactam; TZP, piperacillin-tazobactam; CZA, ceftazidime-avibactam; ATM, aztreonam; CIP, ciprofloxacin; LEV, levofloxacin; AMK, amikacin; TGC, tigecycline; POLB, Polymixin B; SXT, trimethoprim-sulfamethoxazole. POLB, / according to recent CLSI breakpoints of POLB, eliminated the susceptible category and moved susceptible to intermediate and SXT no intermediate category breakpoints. Interpretations of susceptibilities were assigned according to the Clinical and Laboratory Standards Institute (CLSI M100-ED31) guideline. Tigecycline susceptibility was followed based on FDA *Enterobacterales* breakpoints. S, susceptible; I, intermediate; R, resistant.

| **Antibiotics** | **Total** | | | | | **IC2 (%)** | | | | | **IC2A (%)** | | | | |
| --- | --- | --- | --- | --- | --- | --- | --- | --- | --- | --- | --- | --- | --- | --- | --- |
|  | **n=100** | | | | | **n=92** | | | | | **n=38** | | | | |
|  | **R** | **I** | **S** | **MIC50** | **MIC90** | **R** | **I** | **S** | **MIC50** | **MIC90** | **R** | **I** | **S** | **MIC50** | **MIC90** |
| **TZP** | 100.0 (100/100) | 0.0 (0/100) | 0.0 (0/100) | ≥256/4 | ≥256/4 | 100 | 0 | 0 | ≥256/4 | ≥256/4 | 100 | 0 | 0 | ≥256/4 | ≥256/4 |
| **CRO** | 100.0 (100/100) | 0.0 (0/100) | 0.0 (0/100) | ≥32 | ≥32 | 100 | 0 | 0 | ≥32 | ≥32 | 100 | 0 | 0 | ≥32 | ≥32 |
| **CAZ** | 100.0 (100/100) | 0.0 (0/100) | 0.0 (0/100) | ≥32 | ≥32 | 100 | 0 | 0 | ≥32 | ≥32 | 100 | 0 | 0 | ≥32 | ≥32 |
| **CSF** | 99.0 (99/100) | 0.0 (0/100) | 1.0 (1/100) | 128/64 | ≥128/64 | 98.9 | 0 | 1.1 | 128/64 | ≥128/64 | 100 | 0 | 0 | 64/32 | ≥128/64 |
| **FEP** | 97.0 (97/100) | 2.0 (2/100) | 1.0 (1/100) | 128 | ≥128 | 96.7 | 2.2 | 1.1 | 128 | ≥128 | 97.4 | 0 | 2.6 | 128 | ≥128 |
| **IPM** | 100.0 (100/100) | 0.0 (0/100) | 0.0 (0/100) | 64 | 128 | 100 | 0 | 0 | 64 | ≥64 | 100 | 0 | 0 | 64 | 128 |
| **MEM** | 100.0 (100/100) | 0.0 (0/100) | 0.0 (0/100) | 64 | ≥64 | 100 | 0 | 0 | 64 | 128 | 100 | 0 | 0 | 64 | ≥64 |
| **CZA** | 88.0 (88/100) | 8.0 (8/100) | 4.0 (4/100) | 23468 | ≥64/4 | 87 | 8.7 | 4.3 | 23468 | ≥64/4 | 97.4 | 0 | 2.6 | 23468 | ≥64/4 |
| **CIP** | 100.0 (100/100) | 0.0 (0/0) | 0.0(0/0) | ≥8 | ≥8 | 100 | 0 | 0 | ≥8 | ≥8 | 100 | 0 | 0 | ≥8 | ≥8 |
| **LEV** | 93.0 (93/100) | 7.0 (7/100) | 0.0 (0/0) | 16 | ≥16 | 92.4 | 7.6 | 0 | 16 | ≥16 | 89.5 | 10.5 | 0 | 16 | ≥16 |
| **AMK** | 76.0 (76/100) | 1.0 (1/100) | 23.0 (23/100) | ≥128 | ≥128 | 78.3 | 1.1 | 20.7 | ≥128 | ≥128 | 68.4 | 0 | 31.6 | ≥128 | ≥128 |
| **TGC** | 7.0 (7/100) | 11.0 (11/100) | 82.0 (88/100) | 1 | 4 | 7.6 | 10.9 | 81.5 | 1 | 4 | 7.9 | 10.5 | 81.6 | 1 | 4 |
| **POLB** | 5.0 (5/100) | 95.0 (95/100) | / | 0.5 | 1 | 5.4 | 94.6 | / | 0.5 | 1 | 5.3 | 94.7 | / | 0.5 | 1 |
| **SXT** | 91.0 (91/100) | / | 9.0 (9/100) | ≥32/608 | ≥32/608 | 92.4 | / | 7.6 | ≥32/608 | ≥32/608 | 92.1 | / | 7.9 | ≥32/608 | ≥32/608 |

| **Antibiotics** | | | **IC2B (%)** | | | | **IC2C (%)** | | | | | **IC11 (%)** | | | |
| --- | --- | --- | --- | --- | --- | --- | --- | --- | --- | --- | --- | --- | --- | --- | --- |
|  | **n=35** | | | | | **n=19** | | | | | **n=8** | | | | |
|  | **R** | **I** | **S** | **MIC50** | **MIC90** | **R** | **I** | **S** | **MIC50** | **MIC90** | **R** | **I** | **S** | **MIC50** | **MIC90** |
| **TZP** | 100 | 0 | 0 | ≥256/4 | ≥256/4 | 100 | 0 | 0 | ≥256/4 | ≥256/4 | 100 | 0 | 0 | ≥256/4 | ≥256/4 |
| **CRO** | 100 | 0 | 0 | ≥32 | ≥32 | 100 | 0 | 0 | ≥32 | ≥32 | 100 | 0 | 0 | ≥32 | ≥32 |
| **CAZ** | 100 | 0 | 0 | ≥32 | ≥32 | 100 | 0 | 0 | ≥32 | ≥32 | 100 | 0 | 0 | ≥32 | ≥32 |
| **CSF** | 100 | 0 | 0 | 128/64 | ≥128/64 | 94.7 | 0 | 5.3 | 128/64 | ≥128/64 | 100 | 0 | 0 | ≥128/64 | ≥128/64 |
| **FEP** | 97.1 | 2.9 | 0 | 128 | ≥128 | 94.7 | 5.3 | 0 | 64 | ≥128 | 100 | 0 | 0 | 128 | ≥128 |
| **IPM** | 100 | 0 | 0 | 64 | 128 | 100 | 0 | 0 | 64 | 64 | 100 | 0 | 0 | 128 | ≥128 |
| **MEM** | 100 | 0 | 0 | 64 | ≥64 | 100 | 0 | 0 | 64 | ≥64 | 100 | 0 | 0 | ≥64 | ≥64 |
| **CZA** | 94.3 | 5.7 | 0 | 23468 | ≥64/4 | 52.6 | 31.6 | 15.8 | 11780 | ≥64/4 | 100 | 0 | 0 | ≥64/4 | ≥64/4 |
| **CIP** | 100 | 0 | 0 | ≥8 | ≥8 | 100 | 0 | 0 | ≥8 | ≥8 | 100 | 0 | 0 | ≥8 | ≥8 |
| **LEV** | 94.3 | 5.7 | 0 | 16 | ≥16 | 100 | 0 | 0 | 16 | ≥16 | 100 | 0 | 0 | 16 | ≥16 |
| **AMK** | 85.7 | 0 | 14.3 | ≥128 | ≥128 | 84.2 | 5.3 | 10.5 | ≥128 | ≥128 | 50 | 0 | 50 | 16 | ≥128 |
| **TGC** | 11.4 | 8.6 | 80 | 1 | 8 | 0 | 15.8 | 84.2 | 1 | 4 | 0 | 12.5 | 87.5 | 1 | 2 |
| **POLB** | 8.6 | 91.4 | / | 0.5 | 2 | 0 | 100 | / | 0.5 | 0.5 | 0 | 100 | / | 0.5 | 0.5 |
| **SXT** | 94.3 | / | 5.7 | ≥32/608 | ≥32/608 | 94.7 | / | 5.3 | ≥32/608 | ≥32/608 | 50 | / | 50 | 2/38 | ≥32/608 |

**Table S4. Plasmids within each sublineage CRAB**

**R3-T1 (formerly RepAci1 corresponding to GR2) replicon plasmids:** R3-T1 pSRM1.2 and R3-T1 pYFY24.2 exhibit identical genome sequences. **RP-T1 (formerly RepAci6 corresponding to GR6) replicon plasmids:** RP-T1 pYFY21.1 and RP-T1 pYFY24.1 have nearly identical genome sequences with approximately 97.9% nucleotide identity, showing roughly 70,774 bp overlap. Both of them show approximately 64,840 bp overlap between RP-T1 pSRM1.1 with 84.9% nucleotide identity. *The *bla*_OXA-23_-containing Tn*2006* within AbaR4 is inserted upstream of plasmid maintenance region in RP-T1 pSRM1.1. **R3-T3 (GR24) replicon plasmids:** R3-T3 pYFY27.1 shows approximately 101,306 bp alignment with R3-T3 pSRM21.1, harboring 85.54% nucleotide identity. **R3-T4 (formerly RepAci9 corresponding to GR8) plasmids:** R3-T4 pYFY3.1 and R3-T4 pSRM25.1 have identical genome sequences, sharing 76.9% nucleotide identity with R3-T4 pYFY27.2. **R1-T2 (GR16) plasmid:** R1-T2 pSRM3.2. The complete sequence of pSRM3.2 is identical to the reference sequence pDETABR21-5 (GenBank accession number CP088900.1). **NA:** pSRM3.1, pSRM3.3 and pSRM3.4 do not contain a recognizable *rep* gene and therefore cannot be assigned to a Rep group according to established typing protocols for *Acinetobacter* plasmids. The complete sequences of pSRM3.1, pSRM3.3 and pSRM3.4 were identical to pDETABR21-1 (GenBank accession number CP088896.1), pDETABR21-2 (GenBank accession number CP088897.1) and pDETABR21-4 (GenBank accession number CP088899.1) of reference strain DETAB-R21 deposited in the NCBI database.

| **Plasmid** | **Size** | **Type (*rep* group)** | **Resistance genes** | **Reference from** | | | **GenBank accession** |
| --- | --- | --- | --- | --- | --- | --- | --- |
|  |  |  |  | **Sublineage** | **cgST** | **Isolate** |  |
| **pYFY3.1** | 11.19kb | R3-T4 (GR8_RepAci9) | none | IC2A | 668 | YFY3 | CP144256 |
| **pSRM25.1** | 11.19kb | R3-T4 (GR8_RepAci9) | none |  | 596 | SRM25 | CP144241 |
| **pSRM1.2** | 8.73kb | R3-T1 (GR2_RepAci1) | none | IC2B | 906 | SRM1 | CP152385   CP152384 |
| **pSRM1.1** | 86.32kb | RP-T1 (GR6_RepAci6) | *bla*_OXA-23*_ |  |  |  |  |
| **pYFY24.2** | 8.73kb | R3-T1 (GR2_RepAci1) | none |  | 906 | YFY24 | CP144251  CP144250 |
| **pYFY24.1** | 72.25kb | RP-T1 (GR6_RepAci6) | none |  |  |  |  |
| **pYFY21.1** | 72.25kb | RP-T1 (GR6_RepAci6) | none | IC2C | 923 | YFY21 | CP144248 |
| **pSRM21.1** | 112.15kb | R3-T3 (GR24) | none |  | 661 | SRM21 | CP144239 |
| **pYFY27.1** | 112.52kb | R3-T3 (GR24) | none |  | 909 | YFY27 | CP144253  CP144254 |
| **pYFY27.2** | 11.15 kb | R3-T4 (GR8_RepAci9) | none |  |  |  |  |
| **pSRM3.1** | 12.70kb | NA | none | IC11 | 1101 | SRM3 | CP144243  CP144244  CP144245  CP144246 |
| **pSRM3.2** | 2.3kb | R1-T2 (GR16) | none |  |  |  |  |
| **pSRM3.3** | 4.5kb | NA | none |  |  |  |  |
| **pSRM3.4** | 2.7kb | NA | none |  |  |  |  |

**Table S5. Characteristics of IC11 (ST164^Pas^/ST1418^Ox^) *A. baumannii* (28 Pathogenwatch assemblies, 8 assemblies in this study and 2 GenBank complete genomes).** /**:** not available

**Table S6. Characteristics of 92 OXA-23-producing IC2 *A. baumannii* in this study. International clone (IC)**

| **Strain** | **ST^Pas^** | **ST^Ox^** | **Sublineage** | **cgST** | **Carbs** | **Transposon** | **Resource** | **assembly level** | **Platform** |
| --- | --- | --- | --- | --- | --- | --- | --- | --- | --- |
| EFY1 | ST2 | 208 | IC2A | 596 | *bla*_OXA-66_, *bla*_OXA-23_ | Tn*2009* | This study | Contig | Illumina |
| EFY11 | ST2 | 540 | IC2C | 923 | *bla*_OXA-66_, *bla*_OXA-23_ | Tn*2009* | This study | Contig | Illumina |
| EFY12 | ST2 | 540 | IC2C | 923 | *bla*_OXA-66_, *bla*_OXA-23_ | Tn*2009* | This study | Contig | Illumina |
| EFY13 | ST2 | 540 | IC2C | 923 | *bla*_OXA-66_, *bla*_OXA-23_ | Tn*2009* | This study | Contig | Illumina |
| EFY14 | ST2 | 540 | IC2C | 923 | *bla*_OXA-66_, *bla*_OXA-23_ | Tn*2009* | This study | Contig | Illumina |
| EFY15 | ST2 | 136 | IC2B | 906 | *bla*_OXA-66_, *bla*_OXA-23_ | Tn*2006* | This study | Contig | Illumina |
| EFY16 | ST2 | 208 | IC2A | 596 | *bla*_OXA-66_, *bla*_OXA-23_ | Tn*2009* | This study | Contig | Illumina |
| EFY17 | ST2 | 208 | IC2A | 596 | *bla*_OXA-66_, *bla*_OXA-23_ | Tn*2009* | This study | Contig | Illumina |
| EFY18 | ST2 | 208 | IC2A | 668 | *bla*_OXA-66_, *bla*_OXA-23_ | Tn*2009* | This study | Contig | Illumina |
| EFY19 | ST2 | 136 | IC2A | 668 | *bla*_OXA-66_, *bla*_OXA-23_ | Tn*2009* | This study | Contig | Illumina |
| EFY2 | ST2 | 136 | IC2B | 906 | *bla*_OXA-66_, *bla*_OXA-23_ | Tn*2006* | This study | Contig | Illumina |
| EFY20 | ST2 | 540 | IC2C | 923 | *bla*_OXA-66_, *bla*_OXA-23_ | Tn*2009* | This study | Contig | Illumina |
| EFY21 | ST2 | 208 | IC2A | 596 | *bla*_OXA-66_, *bla*_OXA-23_ | Tn*2009* | This study | Contig | Illumina |
| EFY22 | ST2 | 208 | IC2A | 596 | *bla*_OXA-66_, *bla*_OXA-23_ | Tn*2009* | This study | Contig | Illumina |
| EFY23 | ST2 | 208 | IC2A | 596 | *bla*_OXA-66_, *bla*_OXA-23_ | Tn*2009* | This study | Contig | Illumina |
| EFY24 | ST2 | 208 | IC2A | 596 | *bla*_OXA-66_, *bla*_OXA-23_ | Tn*2009* | This study | Contig | Illumina |
| EFY25 | ST2 | 540 | IC2C | 923 | *bla*_OXA-66_, *bla*_OXA-23_ | Tn*2009* | This study | Contig | Illumina |
| EFY26 | ST2 | 195 | IC2B | 906 | *bla*_OXA-66_, *bla*_OXA-23_ | Tn*2006* | This study | Contig | Illumina |
| EFY27 | ST2 | 208 | IC2A | 596 | *bla*_OXA-66_, *bla*_OXA-23_ | Tn*2009* | This study | Contig | Illumina |
| EFY28 | ST2 | 208 | IC2A | 596 | *bla*_OXA-66_, *bla*_OXA-23_ | Tn*2009* | This study | Contig | Illumina |
| EFY29 | ST2 | 208 | IC2C | 909 | *bla*_OXA-66_, *bla*_OXA-23_ | Tn*2009* | This study | Contig | Illumina |
| EFY3 | ST2 | 136 | IC2B | 906 | *bla*_OXA-66_, *bla*_OXA-23_ | Tn*2006* | This study | Contig | Illumina |
| EFY30 | ST2 | 208 | IC2A | 596 | *bla*_OXA-66_, *bla*_OXA-23_ | Tn*2009* | This study | Contig | Illumina |
| EFY31 | ST2 | 208 | IC2A | 596 | *bla*_OXA-66_, *bla*_OXA-23_ | Tn*2009* | This study | Contig | Illumina |
| EFY32 | ST2 | 208 | IC2A | 596 | *bla*_OXA-66_, *bla*_OXA-23_ | Tn*2009* | This study | Contig | Illumina |
| EFY33 | ST2 | 540 | IC2C | 923 | *bla*_OXA-66_, *bla*_OXA-23_ | Tn*2009* | This study | Contig | Illumina |
| EFY34 | ST2 | 208 | IC2A | 668 | *bla*_OXA-66_, *bla*_OXA-23_ | Tn*2009* | This study | Contig | Illumina |
| EFY35 | ST2 | 208 | IC2A | 596 | *bla*_OXA-66_, *bla*_OXA-23_ | Tn*2009* | This study | Contig | Illumina |
| EFY5 | ST2 | 136 | IC2B | 906 | *bla*_OXA-66_, *bla*_OXA-23_ | Tn*2006* | This study | Contig | Illumina |
| EFY6 | ST2 | 136 | IC2B | 906 | *bla*_OXA-66_, *bla*_OXA-23_ | Tn*2006* | This study | Contig | Illumina |
| EFY7 | ST2 | 208 | IC2C | 909 | *bla*_OXA-66_, *bla*_OXA-23_ | Tn*2009* | This study | Contig | Illumina |
| EFY8 | ST2 | 208 | IC2A | 596 | *bla*_OXA-66_, *bla*_OXA-23_ | Tn*2009* | This study | Contig | Illumina |
| EFY9 | ST2 | 540 | IC2C | 923 | *bla*_OXA-66_, *bla*_OXA-23_ | Tn*2009* | This study | Contig | Illumina |
| SRM1 | ST2 | 136 | IC2B | 906 | *bla*_OXA-66_, *bla*_OXA-23_ | Tn*2006* | This study | Complete genome | Illumina, Nanopore |
| SRM10 | ST2 | 208 | IC2A | 596 | *bla*_OXA-66_, *bla*_OXA-23_ | Tn*2009* | This study | Contig | Illumina |
| SRM11 | ST2 | 208 | IC2B | 906 | *bla*_OXA-66_, *bla*_OXA-23_ | Tn*2006* | This study | Contig | Illumina |
| SRM12 | ST2 | 208 | IC2B | 906 | *bla*_OXA-66_, *bla*_OXA-23_ | Tn*2006* | This study | Contig | Illumina |
| SRM13 | ST2 | 208 | IC2B | 906 | *bla*_OXA-66_, *bla*_OXA-23_ | Tn*2006* | This study | Contig | Illumina |
| SRM14 | ST2 | 208 | IC2B | 906 | *bla*_OXA-66_, *bla*_OXA-23_ | Tn*2006* | This study | Contig | Illumina |
| SRM15 | ST2 | 195 | IC2B | 906 | *bla*_OXA-66_, *bla*_OXA-23_ | Tn*2006* | This study | Contig | Illumina |
| SRM16 | ST2 | 208 | IC2B | 906 | *bla*_OXA-66_, *bla*_OXA-23_ | Tn*2006* | This study | Contig | Illumina |
| SRM17 | ST2 | 195 | IC2B | 906 | *bla*_OXA-66_, *bla*_OXA-23_ | Tn*2006* | This study | Contig | Illumina |
| SRM18 | ST2 | 2499 | IC2A | 596 | *bla*_OXA-66_, *bla*_OXA-23_ | Tn*2009* | This study | Contig | Illumina |
| SRM19 | ST2 | 208 | IC2B | 906 | *bla*_OXA-66_, *bla*_OXA-23_ | Tn*2006* | This study | Contig | Illumina |
| SRM2 | ST2 | 208 | IC2A | 596 | *bla*_OXA-66_, *bla*_OXA-23_ | Tn*2009* | This study | Contig | Illumina |
| SRM20 | ST2 | 208 | IC2C | 661 | *bla*_OXA-66_, *bla*_OXA-23_ | Tn*2009* | This study | Contig | Illumina |
| SRM21 | ST2 | 208 | IC2C | 661 | *bla*_OXA-66_, *bla*_OXA-23_ | Tn*2009* | This study | Complete genome | Illumina, Nanopore |
| SRM22 | ST2 | 208 | IC2A | 596 | *bla*_OXA-66_, *bla*_OXA-23_ | Tn*2009* | This study | Contig | Illumina |
| SRM23 | ST2 | 208 | IC2A | 596 | *bla*_OXA-66_, *bla*_OXA-23_ | Tn*2009* | This study | Contig | Illumina |
| SRM25 | ST2 | 2499 | IC2A | 596 | *bla*_OXA-66_, *bla*_OXA-23_ | Tn*2009* | This study | Complete genome | Illumina, Nanopore |
| SRM26 | ST2 | 195 | IC2B | 906 | *bla*_OXA-66_, *bla*_OXA-23_ | Tn*2006* | This study | Contig | Illumina |
| SRM27 | ST2 | 195 | IC2B | 906 | *bla*_OXA-66_, *bla*_OXA-23_ | Tn*2006* | This study | Contig | Illumina |
| SRM29 | ST2 | 195 | IC2B | 906 | *bla*_OXA-66_, *bla*_OXA-23_ | Tn*2006* | This study | Contig | Illumina |
| SRM30 | ST2 | 540 | IC2C | 923 | *bla*_OXA-66_, *bla*_OXA-23_ | Tn*2009* | This study | Contig | Illumina |
| SRM31 | ST2 | 208 | IC2A | 596 | *bla*_OXA-66_, *bla*_OXA-23_ | Tn*2009* | This study | Contig | Illumina |
| SRM32 | ST2 | 136 | IC2B | 906 | *bla*_OXA-66_, *bla*_OXA-23_ | Tn*2006* | This study | Contig | Illumina |
| SRM33 | ST2 | 195 | IC2B | 906 | *bla*_OXA-66_, *bla*_OXA-23_ | Tn*2006* | This study | Contig | Illumina |
| SRM34 | ST2 | 208 | IC2A | 596 | *bla*_OXA-66_, *bla*_OXA-23_ | Tn*2009* | This study | Contig | Illumina |
| SRM35 | ST2 | 540 | IC2C | 923 | *bla*_OXA-66_, *bla*_OXA-23_ | Tn*2009* | This study | Contig | Illumina |
| SRM36 | ST2 | 369 | IC2B | 906 | *bla*_OXA-66_, *bla*_OXA-23_ | Tn*2006* | This study | Contig | Illumina |
| SRM37 | ST2 | 208 | IC2A | 596 | *bla*_OXA-66_, *bla*_OXA-23_ | Tn*2009* | This study | Contig | Illumina |
| SRM4 | ST2 | 208 | IC2A | 596 | *bla*_OXA-66_, *bla*_OXA-23_ | Tn*2009* | This study | Contig | Illumina |
| SRM5 | ST2 | 208 | IC2B | 906 | *bla*_OXA-66_, *bla*_OXA-23_ | Tn*2006* | This study | Contig | Illumina |
| SRM6 | ST2 | 208 | IC2B | 906 | *bla*_OXA-66_, *bla*_OXA-23_ | Tn*2006* | This study | Contig | Illumina |
| SRM7 | ST2 | 208 | IC2A | 596 | *bla*_OXA-66_, *bla*_OXA-23_ | Tn*2009* | This study | Contig | Illumina |
| SRM8 | ST2 | 208 | IC2B | 906 | *bla*_OXA-66_, *bla*_OXA-23_ | Tn*2006* | This study | Contig | Illumina |
| YFY1 | ST2 | 208 | IC2A | 596 | *bla*_OXA-66_, *bla*_OXA-23_ | Tn*2009* | This study | Contig | Illumina |
| YFY11 | ST2 | 208 | IC2A | 596 | *bla*_OXA-66_, *bla*_OXA-23_ | Tn*2009* | This study | Contig | Illumina |
| YFY12 | ST2 | 381 | IC2A | 668 | *bla*_OXA-66_, *bla*_OXA-23_ | Tn*2009* | This study | Contig | Illumina |
| YFY13 | ST2 | 208 | IC2B | 906 | *bla*_OXA-66_, *bla*_OXA-23_ | Tn*2006* | This study | Contig | Illumina |
| YFY14 | ST2 | 195 | IC2B | 906 | *bla*_OXA-66_, *bla*_OXA-23_ | Tn*2006* | This study | Contig | Illumina |
| YFY16 | ST2 | 136 | IC2B | 906 | *bla*_OXA-66_, *bla*_OXA-23_ | Tn*2006* | This study | Contig | Illumina |
| YFY17 | ST2 | 381 | IC2A | 668 | *bla*_OXA-66_, *bla*_OXA-23_ | Tn*2009* | This study | Contig | Illumina |
| YFY18 | ST2 | 208 | IC2B | 906 | *bla*_OXA-66_, *bla*_OXA-23_ | Tn*2006* | This study | Contig | Illumina |
| YFY19 | ST2 | 208 | IC2B | 906 | *bla*_OXA-66_, *bla*_OXA-23_ | Tn*2006* | This study | Contig | Illumina |
| YFY2 | ST2 | 208 | IC2B | 906 | *bla*_OXA-66_, *bla*_OXA-23_ | Tn*2006* | This study | Contig | Illumina |
| YFY20 | ST2 | 208 | IC2A | 596 | *bla*_OXA-66_, *bla*_OXA-23_ | Tn*2009* | This study | Contig | Illumina |
| YFY21 | ST2 | 540 | IC2C | 923 | *bla*_OXA-66_, *bla*_OXA-23_ | Tn*2009* | This study | Complete genome | Illumina, Nanopore |
| YFY22 | ST2 | 136 | IC2B | 906 | *bla*_OXA-66_, *bla*_OXA-23_ | Tn*2006* | This study | Contig | Illumina |
| YFY23 | ST2 | 208 | IC2A | 596 | *bla*_OXA-66_, *bla*_OXA-23_ | Tn*2009* | This study | Contig | Illumina |
| YFY24 | ST2 | 136 | IC2B | 906 | *bla*_OXA-66_, *bla*_OXA-23_ | Tn*2006* | This study | Complete genome | Illumina, Nanopore |
| YFY25 | ST2 | 195 | IC2B | 906 | *bla*_OXA-66_, *bla*_OXA-23_ | Tn*2006* | This study | Contig | Illumina |
| YFY26 | ST2 | 208 | IC2A | 596 | *bla*_OXA-66_, *bla*_OXA-23_ | Tn*2009* | This study | Contig | Illumina |
| YFY27 | ST2 | 208 | IC2C | 909 | *bla*_OXA-66_, *bla*_OXA-23_ | Tn*2009* | This study | Complete genome | Illumina, Nanopore |
| YFY28 | ST2 | 195 | IC2B | 906 | *bla*_OXA-66_, *bla*_OXA-23_ | Tn*2006* | This study | Contig | Illumina |
| YFY29 | ST2 | 208 | IC2C | 923 | *bla*_OXA-66_, *bla*_OXA-23_ | Tn*2009* | This study | Contig | Illumina |
| YFY3 | ST2 | 381 | IC2A | 668 | *bla*_OXA-66_, *bla*_OXA-23_ | Tn*2009* | This study | Complete genome | Illumina, Nanopore |
| YFY4 | ST2 | 540 | IC2C | 923 | *bla*_OXA-66_, *bla*_OXA-23_ | Tn*2009* | This study | Contig | Illumina |
| YFY6 | ST2 | 540 | IC2C | 923 | *bla*_OXA-66_, *bla*_OXA-23_ | Tn*2009* | This study | Contig | Illumina |
| YFY7 | ST2 | 195 | IC2B | 906 | *bla*_OXA-66_, *bla*_OXA-23_ | Tn*2006* | This study | Contig | Illumina |
| YFY8 | ST2 | 381 | IC2A | 668 | *bla*_OXA-66_, *bla*_OXA-23_ | Tn*2009* | This study | Contig | Illumina |
| YFY9 | ST2 | 208 | IC2A | 596 | *bla*_OXA-66_, *bla*_OXA-23_ | Tn*2009* | This study | Contig | Illumina |

**Supplementary Figure Legends**

**Fig. S1 Pulsed-field gel electrophoresis (PFGE) characteristics** **of 100 clinical CRAB isolates.** According to the 85% cutoff level defined as clonal clusters using *ApaI* as a restriction enzyme, the 100 CRAB isolates exhibited 11 distinct PFGE profiles, designated as A-K types**.** The main PFGE clone of CRAB in Hospital EFY was the A type, E type and F type. The dominant types of Hospital YFY were B and H types. Type C and E was predominant in Hospital SRM. G and D were all found in these three hospitals. ICU, Intensive Care Unit; CM, Cardiovascular Medicine; CMS, Cardiac and macrovascular surgery; GIS, Gastrointestinal Surgery; CTS, Department of Cardiothoracic Surgery; HS, Hepatobiliary Surgery; Nep, Nephrology; NL, Neurology; NS, Neurosurgery; PCCM, Pulmonary and Critical Care Medicine; EM, Emergency Medicine; RH, Rehabilitation; ULS, Urology; OD, Oncology; ORTH, Orthopedic; GS, General Surgery; CSF, Cerebrospinal Fluid; SP, sputum; BALF, bronchoalveolar lavage fluid; Yes, patients affected CRAB death; NO, patient alive; NA, unknown.


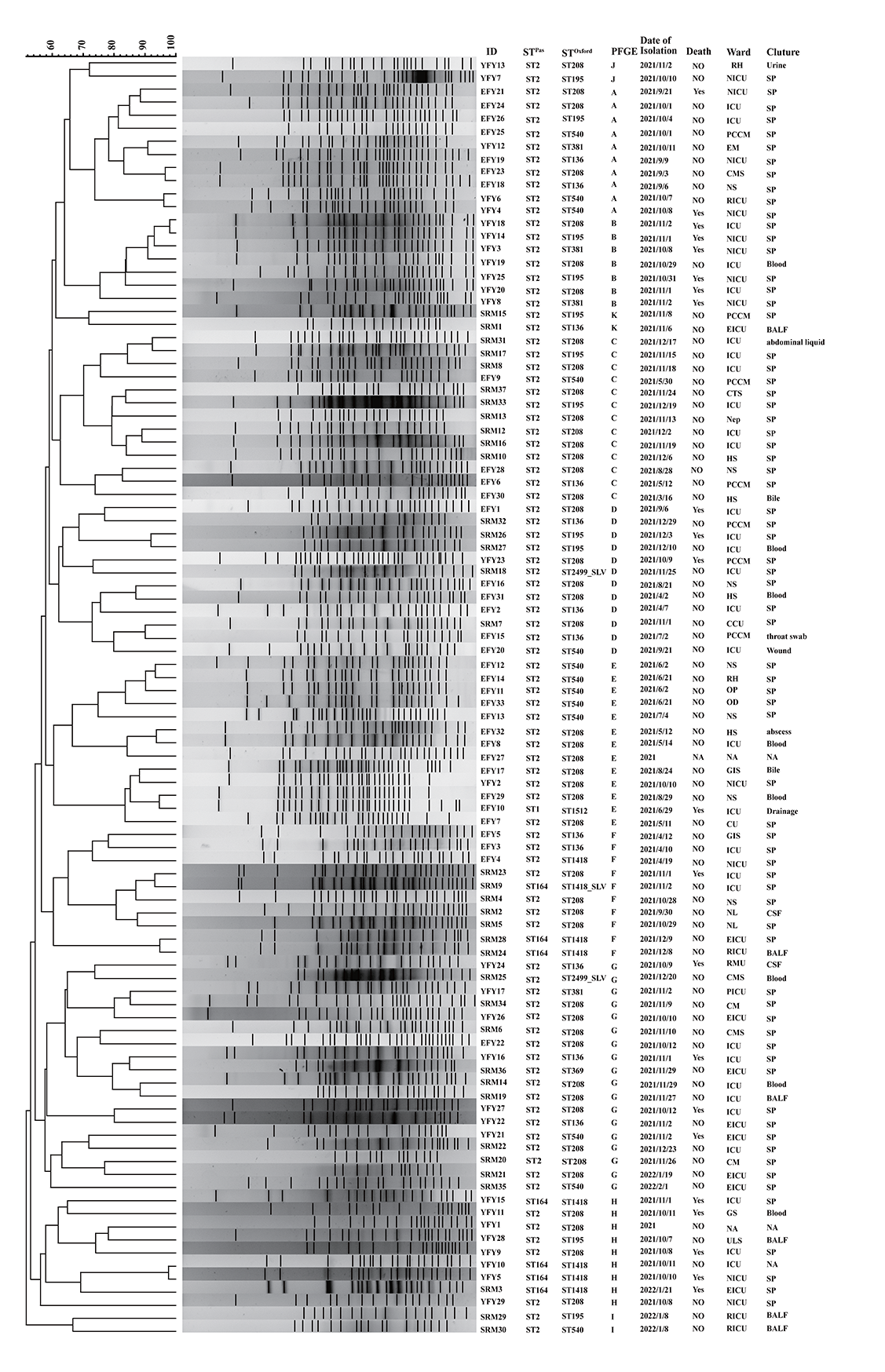
**Fig. S1**
